# Philadelphia College of Medicine

**Published:** 1847-04

**Authors:** 


### ARTICLE VI.

### PHILADELPHIA COLLEGE OF MEDICINE.

This, the fifth medical School in the city of brotherly love, was chartered on the 14th of January last, by the Legislature of Pennsylvania, without a dissenting vote; and so early as the 15th of March, the first course of lectures was commenced. The faculty is as follows:

Thos. D. Mitchell, M. D., Prof. of Theory and Practice, Midwifery and Med. Jurisprudence.

Wm. H. Allen, A. M., Prof. of Chemistry.

J. R. Burden, M. D., Prof. of Mat. Med., and Therapeutics.

James McClintock, M. D., Prof. of Anatomy, Physiology and Surgery.

S. Gore White, M. D., Demonstrator of Anatomy.

